# Polypharmacology of ambroxol in the treatment of COVID-19

**DOI:** 10.1042/BSR20221927

**Published:** 2023-02-27

**Authors:** Ziyuan Wang, Minghui Yang, Xi Chen, Rongxin Xiao, Yu Dong, Ming Chu, Guojie Song, Yuedan Wang

**Affiliations:** 1 Department of Immunology, School of Basic Medical Sciences, Peking University, NHC Key Laboratory of Medical Immunology (Peking University), Beijing, China; 2 The Third Clinical Department of China Medical University, Shenyang, Liaoning, China; 3 Department of Adult Joint Reconstructive Surgery, Beijing Jishuitan Hospital, Fourth Clinical College of Peking University, Jishuitan Orthopaedic College of Tsinghua University, Beijing, China; 4 Department of Thoracic Surgery, Peking University People’s Hospital, Beijing, China; 5 Department of Artificial Intelligence, School of Intelligence Science and Technology, Peking University, Beijing, China

**Keywords:** ambroxol, COVID-19, NF-κB, NRP-1, polypharmacology, SARS-CoV-2

## Abstract

The pandemic of coronavirus disease 2019 (COVID-19) by severe acute respiratory syndrome coronavirus 2 (SARS-CoV-2) is still underway. Due to the growing development of severe symptoms, it is necessary to promote effective therapies. Ambroxol [2-amino-3,5-dibromo-*N*-(*trans*-4-hydroxycyclohexyl) benzylamine] has long been used as one of the over-the-counter mucolytic agents to treat various respiratory diseases. Therefore, we focused on the mechanism of action of ambroxol in COVID-19 treatment. *In vitro* and *in silico* screening revealed that ambroxol may impede cell entry of SARS-CoV-2 by binding to neuropilin-1. Ambroxol could also interact with multiple inflammatory factors and signaling pathways, especially nuclear factor kappa B (NF-κB), to interfere cytokines cascade activated by SARS-CoV-2 internalization. Furthermore, multipathways and proteins, such as the cell cycle and matrix metalloproteinases (MMPs), were identified as significant ambroxol-targeting pathways or molecules in PBMC and lung of severe COVID-19 patients by bioinformatics analysis. Collectively, these results suggested that ambroxol may serve as a promising therapeutic candidate for the treatment of severe SARS-CoV-2 infection.

## Introduction

Since December 2019, a novel viral pneumonia coronavirus disease 2019 (COVID-19), which was caused by severe acute respiratory syndrome coronavirus 2 (SARS-CoV-2), continued to grip the world [1]. As of 9th May 2022, it has spread to more than 200 countries and territories with more than 515 million laboratory-confirmed cases worldwide, resulting in more than 6.3 million deaths since its emergence [2]. The ongoing pandemic has caused widespread economic and social disruption [3]. To prevent further morbidity and death, health organizations over the globe are working to curb the spread of COVID-19 by developing more rapid diagnosis methods for SARS-CoV-2 carriers, as well as efficacious vaccines [4,5].

COVID-19 can be well defined as a disease with two successive phases: an early viral phase characterized by influenza-like upper and lower respiratory tract illness, followed in severe instances by an inflammatory response stage. The latter condition is characterized by inflammation-driven damage to multiorgan system, particularly the lungs, which can result in acute respiratory distress syndrome and life-threatening hypoxia. Serious damage can also occur in other important organs such as the brain and blood vessels [6–8]. While some research indicates that 51.7% of the patients are asymptomatic [9] and most of the infections remain uncontrolled, 13.8% of those infected are critically ill [10].

The World Health Organization recommends systemic corticosteroids, baricitinib, as well as IL-6 receptor blockers for patients with severe or life-threatening COVID-19 [11]. Corticosteroids and long-acting bronchodilators may reduce the replication of coronaviruses, including SARS-CoV-2, and provide a relative 21% reduction in mortality, according to some laboratory evidence [12]. Elevated concentrations of IL-6 are strongly associated with severe COVID-19 outcomes. Thus, IL-6 receptor blockers, such as tocilizumab and sarilumab, can antagonize membrane-bound and soluble forms of the IL-6 receptor, block the cytokine activation, and regulate the immune response upon infection [11]. Despite these therapies, symptomatic treatment approaches remain uncertain. Many patients with severe COVID-19 experience progressive respiratory failure, including bilateral infiltrates and lung edema caused by mucus obstruction [13], which may develop to a cause of death in COVID-19. Given that ambroxol has been used clinically as a first-line drug in expectoration with high dosages of intravenous injection, it is noteworthy that ambroxol plays a vital role in the treatment of severe COVID-19. In addition to its mucoactive function, ambroxol was reported to have anti-inflammatory, antioxidant, antiviral, and antibacterial effects [12]. However, its mechanism, particularly in severe instances of COVID-19, remains unexplained. Here, by examining its pharmacology and mechanism, we investigated the therapeutic application of ambroxol in COVID-19.

## Materials and methods

### Pseudovirus

The pseudotyped HIV lentivirus with the spike glycoprotein of SARS-CoV-2 that contains a Luc reporter gene was purchased from Sino Biological Inc. (Beijing, China).

### Cells

HEK293T (ATCC®CRL-3216™) cells stably expressing hACE2 (HEK293T-hACE2) were generated by following a reported protocol [14]. In brief, lentivirus encoding hACE2 (Sino Biological, Beijing, China) were used to transduce HEK293T cells and selected them for 14 days with 2 μg/ml of puromycin (InvivoGen). The puromycin-resistant cells were maintained in Dulbecco’s modified eagle medium (DMEM; Gibco) supplemented with 10% heat-inactivated fetal bovine serum (FBS; Gibco), 50 U/ml penicillin, 50 μg/ml streptomycin, and 1 μg/ml puromycin. The expression of hACE2 in HEK293T-hACE2 cells was determined by western blot analysis.

### Human proteome chip screening

The HuProt™ Human Proteome chip, which contains 23156 human full-length proteins, was used in the human proteomic chip screening. Recombinant proteins with GST tags were expressed and purified by the eukaryotic expression system. For chip screening, two technical replicates were set for each protein on the chip. The chip was blocked at room temperature for 1 h with blocking solution (3% BSA in pH 7.4 PBS Buffer). The chip was then rinsed three times by 0.1% PBST and dried by centrifugation (500 ***g***, 3 min). After that, the chip was incubated with ambroxol (100 μM, 1% BSA in PBST), biotinylated ambroxol diluent (10 μM, 1% BSA in PBST), and biotin (100 μM) as control at 37°C for 1 h. The preceding steps were repeated to wash the chip followed by a pure water rinse and centrifugation. Finally, the chip was scanned by the GenePix 4000B chip scanner. GenePix Pro v6.0 software was used to read and analyze the collected data.

### Target fishing

A pharmacophore model’s characteristics reflect the mechanism of drug–target interaction. By fitting ambroxol to a panel of pharmacophore models, the Ligand Profiler protocol of Discovery Studio 2021 (DS; BIOVIA-Dassault Systèmes), which is equipped with PharmaDB database, was used to spot the potential targets for ambroxol.

### RNA-seq data aqusition and preparation

The gene expression data of COVID-19 patients and healthy controls were downloaded from NCBI GEO database (lung, GSE182917; PBMC, GSE171110) [15,16]. All analyses were carried out with R Studio software (www.rstudio.com) in the R 4.1.1 environment. Before analysis, the original expression data were converted to transcriptomes per million reads (TPM) and transformed to log2 (TPM + 1) format. Differentially expression genes (DEGs) matrix was generated by R package limma with the Benjamini–Hochberg Method to preform *P-*value correction. Genes with both adjusted *P-*value (adj. *P*)<0.05 and |log2 FC| ≥ 1.5 were considered significantly differentially expressed.

### Enrichment analysis

Gene Ontology (GO) and Kyoto Encyclopedia of Genes and Genomes (KEGG) enrichment analyses were carried out by R package clusterProfiler. Significantly enriched elements were identified as themes with adj. *P*<0.05. KEGG pathways were shown by R package pathview.

### Molecular docking

Molecular docking is a common tool for examination protein–small-molecule interactions, to identify receptor-binding sites, and to predict the pharmacological features of prospective small compounds *in silico* [17]. The molecular docking and visualization between ambroxol and neuropilin-1 (NRP-1; PDB ID: 3i97) [18] was performed following the standard protocol of CDOCKER on DS platform. The 3D structure of human neuropilin-2 (NRP-2) was downloaded from AlphaFold Protein Structure Database (AF-O60462-F1) [19]. In binding free-energy calculation, the Generalized Born model was utilized to account for the influence of the solvent. A 1000-step *in situ* ligand minimization of the NRP-1-ambroxol complex was performed prior to calculation.

### Pseudotype-based neutralization assay

Prior to inoculation with the pseudotyped SARS-CoV-2, the HEK293T-hACE2 cells were treated for 1 h with ambroxol at a concentration gradient ranging from 600 to 1 μM [14]. After 1 h of inoculation in the presence of each drug, the inoculum was removed and fresh medium was added for further culture. The activity of firefly luciferase was measured as a readout of infected cells using the luciferase assay (Promega) for quantitative determination at 48 h post-transduction.

## Results

### Polypharmacology of ambroxol

**Figure 1:**
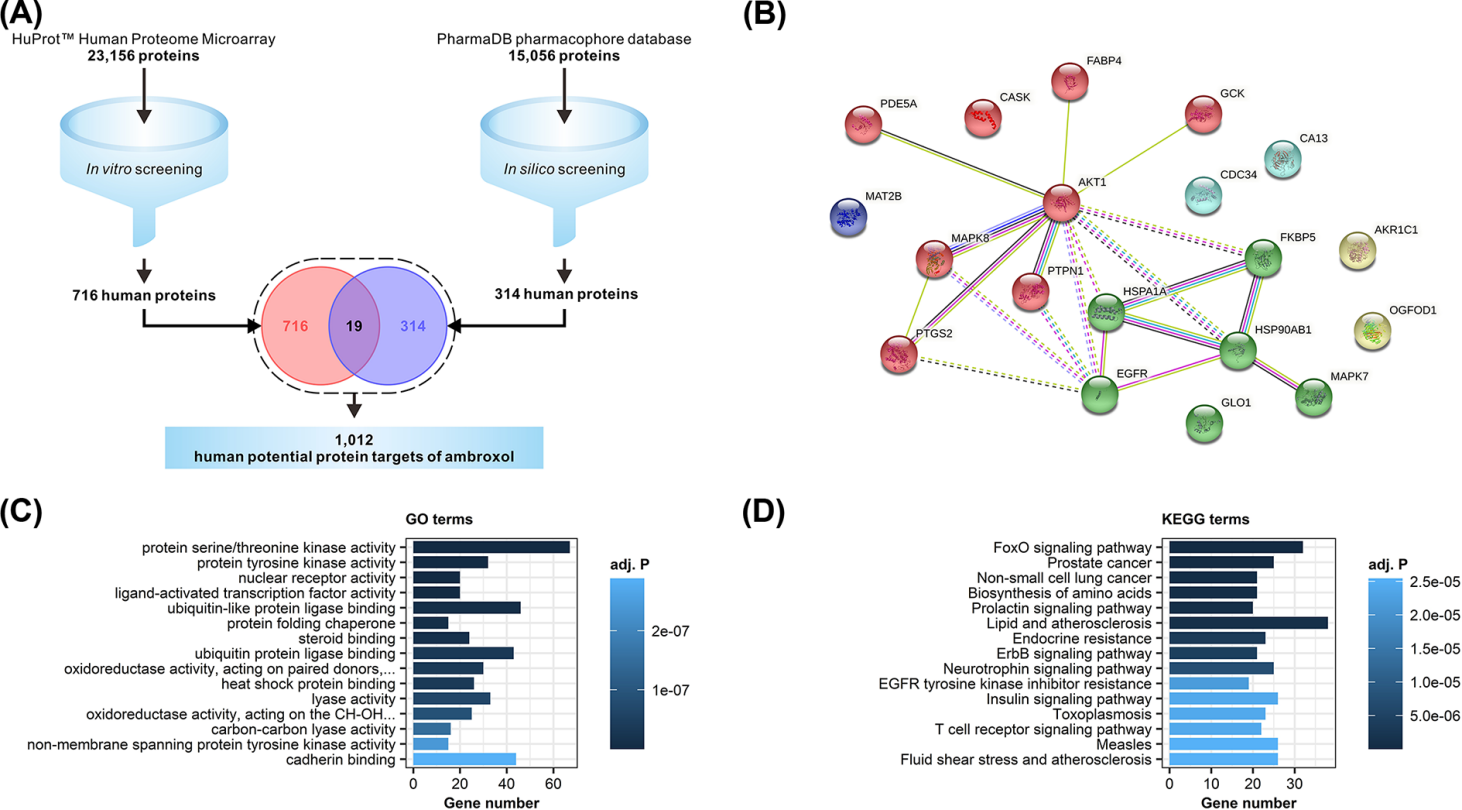
Ambroxol’s polypharmacological profile (**A**) The screening of ambroxol’s potential protein targets in human. Details of the discovery workflow are described in the Methods section. (**B**) PPI network of the intersected targets. The interactions were discovered with a medium confidence rate of 0.4. (**C**) GO enrichment results of potential targets of ambroxol. The top 15 significant themes with adj. *P*<0.05 were shown here. (**D**) KEGG enrichment results of potential targets of ambroxol. The top 15 significant themes with adj. *P*<0.05 were shown here.

In order to discover potential human protein targets of ambroxol, 23156 proteins were obtained from the HuProt™ Human Proteome Microarray, and 716 human proteins were identified via *in vitro* screening. Meanwhile, 15056 proteins extracted from the PharmaDB pharmacophore database were analyzed *in silico* and 314 human proteins were selected (Figure 1A). There were 19 proteins that overlapped between *in vitro* and *in silico* analysis, and these proteins were shown in Table 1. The protein–protein interaction (PPI) network of the intersecting proteins was established, revealing that AKT1 and HSP90AB1 are core molecules in the network (Figure 1B). For further efforts, 1012 potential protein targets were obtained by combine the results together, which were then studied by enrichment analysis. GO annotation suggested that the selected potential targets were mainly associated with intracellular signaling transduction functions such as protein serine/threonine kinase activity, protein tyrosine kinase activity, and ubiquitin-like protein ligase binding (Figure 1C). KEGG annotation indicated that the FoxO signaling pathway was most significantly enriched. In addition, many target genes were strongly associated with cancer-related pathways, such as prostate cancer and non-small-cell lung cancer (NSCLC), suggesting that ambroxol might become a reasonable candidate in antitumor treatment (Figure 1D).

**Table 1:** Overlapped potential targets of ambroxol *in vitro* and *in silico*

Number	Gene name	Chip Z-score	Fit value	PDB ID
1	EGFR	7.917527	0.717333	1M17
2	OGFOD1	5.637994	0.547555	4NHY
3	CASK	5.498071	0.635301	3MFR
4	HSP90AB1	4.982750	0.506164	1UYM
5	FKBP5	4.458578	0.584362	4JFI
6	MAT2B	4.389757	0.607822	2YDX
7	AKR1C1	4.364121	0.595012	1MRQ
8	GLO1	4.217603	0.575641	3W0T
9	PDE5A	4.091471	0.608814	1RKP
10	MAPK8	3.631472	0.554922	2H96
11	PTPN1	3.600931	0.650606	1T48
12	HSPA1A	3.508435	0.585146	5AQZ
13	CA13	3.435829	0.573331	4KNM
14	GCK	3.300854	0.659483	4DCH
15	CDC34	3.242392	0.597149	4MDK
16	PTGS2	3.099950	0.595399	5IKR
17	MAPK7	3.061945	0.537640	4B99
18	FABP4	3.036884	0.577740	1TOW
19	AKT1	2.969269	0.548154	3MV5

**Figure 2:**
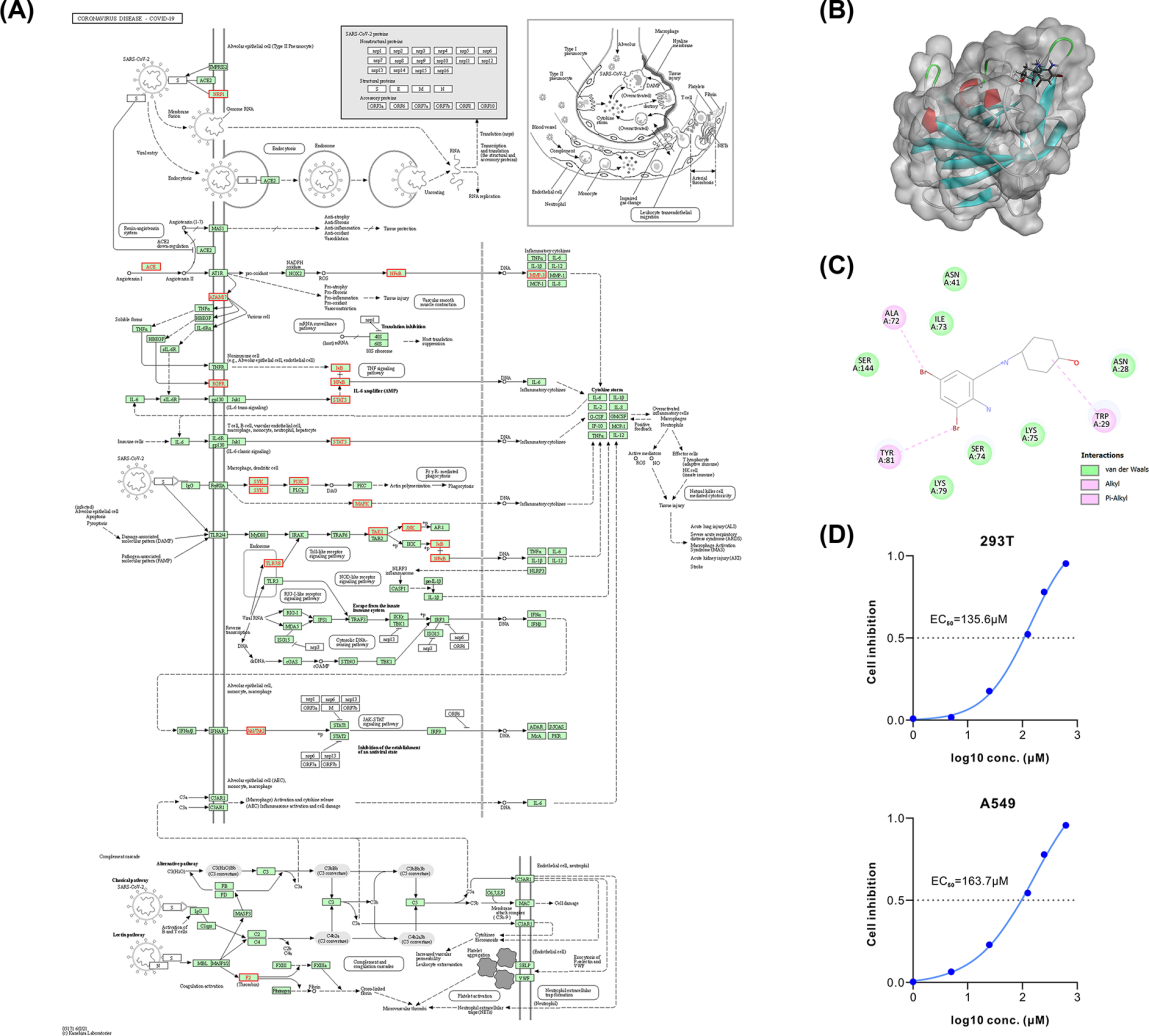
Role of ambroxol in SARS-CoV-2 infection (**A**) A diagram of Coronavirus disease—COVID-19 pathway in KEGG. Proteins shown in the red boxes are the potential targets of ambroxol. (**B**) Possible binding feature of ambroxol with the B1 domain of NRP-1. Ambroxol was shown as sticks with carbon, oxygen, and nitrogen-colored gray, red, and blue, respectively. Secondary structural elements are depicted as ribbons (coils, α-helices; arrows, β-sheets). Color is based on secondary structures (α-helices, red; β-sheets, skyblue; loops, green). (**C**) 2D diagram of molecular interaction of ambroxol with NRP-1. (**D**) Cell inhibition curves of different concentrations of ambroxol in 293T-hACE2 cells and A549 cells.

SARS-CoV-2 infection of a cell triggers cascading signaling transduction pathways that contribute to inflammation and anti-inflammation processes. The mechanism is shown in Figure 2A. Toll-like receptors 7/8 (TLR7/8) can be activated by the viral RNA of SARS-CoV-ACE2, resulting in the activation of the nuclear factor kappa B (NF-κB) signaling cascade. Based on the potential targets identified in our screening, ambroxol might bind to TLR7/8 and subsequent signal conductor proteins in NF-κB/MAPK signaling pathway, including TAK1, NF-κB, I-κB, and MAPK, thus block the signaling pathway at multiple stages. In addition, STAT3 served as the target of ambroxol, suggesting that ambroxol might also inhibit the JAK-STAT signaling pathway. These two pharmacological effects led to the same consequence: a reduction in the production of inflammatory factors including IL-6, IL-1β, IL-8, and TNF-α. In short, ambroxol inhibits SARS-CoV-2 entry and prevents further detrimental inflammatory responses by binding to many primary targets. Therefore, ambroxol is therapeutically applicable for intravenous treatment of COVID-19 infection.

### Role of ambroxol in SARS-CoV-2 infection pathway

By mapping ambroxol’s potential target to Coronavirus disease—COVID-19 pathway in KEGG, we found that ambroxol might interact with the SARS-CoV-2 cell entry receptor, NRP-1, on cellular surface (Figure 2A). We hypothesized that SARS-CoV-2 cell entry process could be impeded by ambroxol through binding to NRP-1. To test the hypothesis, molecular docking was utilized to investigate possible docking conformation between ambroxol and NRP-1. Ambroxol docked into the B1 domain of NRP-1 with a binding energy of −4.4 kcal/mol (Figure 2B,C). We further determined the optimal concentration of ambroxol for inhibiting the entry of SARS-CoV-2 pseudovirus. The concentration for 50% of maximal effect (EC_50_) of ambroxol to inhibit SARS-CoV-2 from entering cells in 293T tool cells expressing high levels of ACE2 was determined to be 135.6 μM (Figure 2D). Additionally, lung adenocarcinoma cells A549 were employed to stimulate alveolar epithelial cells, and we determined that 163.7 μM ambroxol was adequate to prevent cell entry (Figure 2C). As a consequence, we deduced that ambroxol might bind to NRP-1 on cell surface to block SARS-CoV-2 entry.

NRP-2 shared sequence similarity with NRP-1 (Supplementary Figure S1A), and there was evidence that it played a similar role to NRP-1 in the process of SARS-CoV-2-entering cells [20,21]. Molecular docking for ambroxol and NRP-2 revealed that ambroxol is unable to bind to NRP-2 in a similar conformation due to the absence of NRP-1-like active cavity (red circle in Supplementary Figure S1B, also shown in Supplementary Figure S1C), despite NRP-2 being identical to NRP-1 in key amino acid. This decrease the binding free energy of ambroxol to NRP-2 to −1.6 kcal/mol, leading us to assume that the capacity of ambroxol to bind NRP-2 to block viral entrance into cells is inferior to that of NRP-1.

### Polypharmacology of ambroxol in PBMC of severe SARS-CoV-2 patients

**Figure 3:**
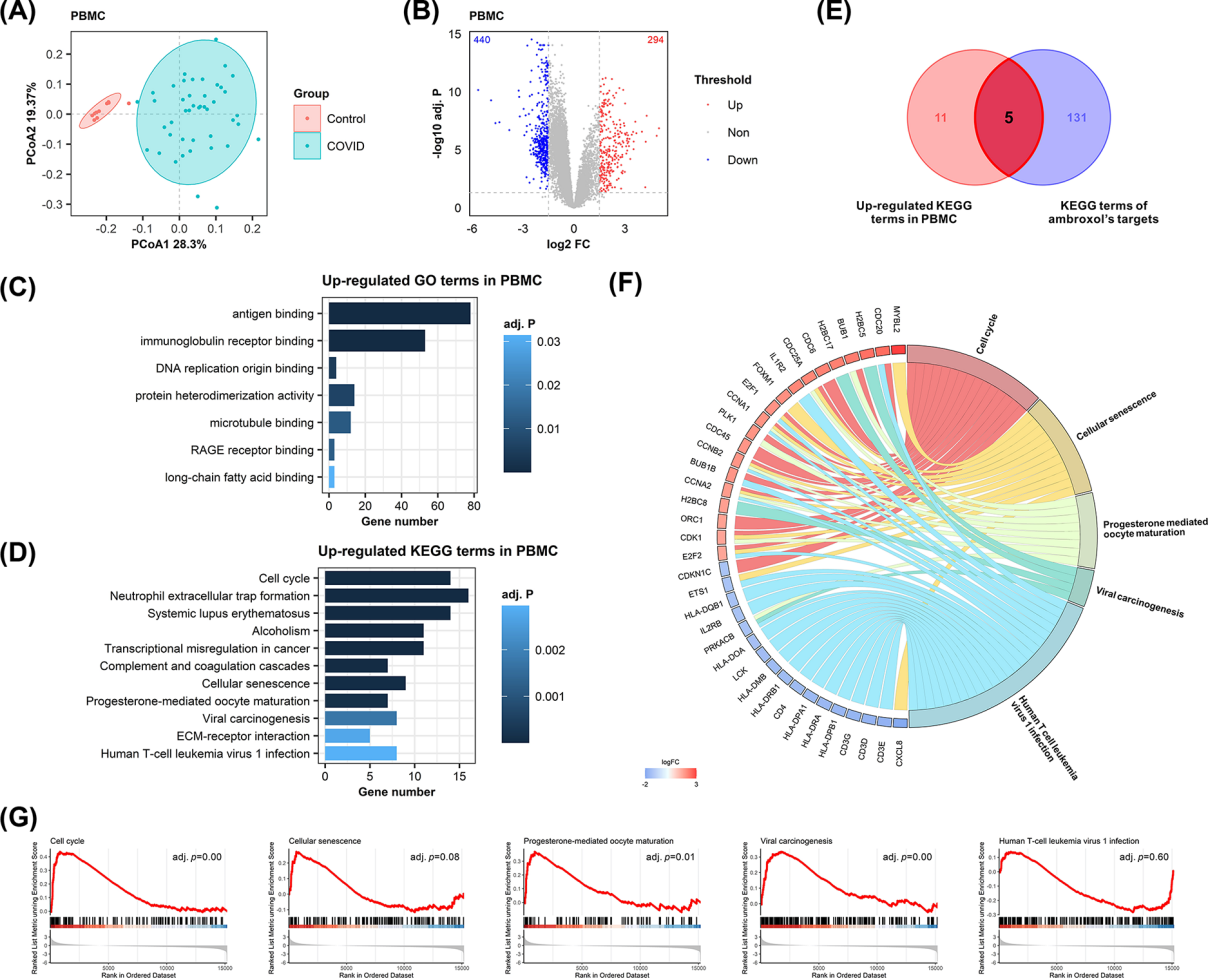
Role of ambroxol in up-regulated pathways in PBMC (**A**) PCoA diagram of differences in PBMC between the COVID-19 patients and the healthy controls. (**B**) Volcano plot of DEGs of PBMC. Genes with adj. *P*<0.05 and |log2FC| > 1 are considered significantly differentially expressed, as shown in upper left and right quadrants. (**C**) GO enrichment results of significantly up-regulated genes in PBMC. The top 15 significant themes with adj. *P*<0.05 were shown here. (**D**) KEGG enrichment results of significantly up-regulated genes in PBMC. The top 15 significant themes with adj. *P*<0.05 were shown here. (**E**) Venn diagram of up-regulated KEGG terms in PBMC and KEGG terms of ambroxol’s targets. (**F**) Chord diagram of up-regulated genes and their corresponding pathways. Color beside gene name indicates range of up-regulate or down-regulate expression. (**G**) GSEA tests of corresponding up-regulated pathways in PBMC.

To evaluate the impact of ambroxol on the PBMC of COVID-19 patients, RNA-seq data from moderate to severe COVID-19 patients and healthy controls were utilized for subsequent analysis. Significant distance difference between the COVID-19 patients and healthy controls was observed via principal coordinates analysis (PCoA) (Figure 3A). The DEGs were further obtained and precented in volcano plot, with 294 significantly up-regulated and 440 down-regulated genes (Figure 3B). GO annotation of the up-regulated genes showed a predominance of associations with immunological processes such as antigen binding, immunoglobulin receptor binding (Figure 3C), and the cell cycle pathway was most significantly enriched in KEGG analysis (Figure 3D). Five pathways intersected between up-regulated pathways and ambroxol’s targets (Figure 3E), which were displayed in Figure 3F with all DEGs. Additionally, GSEA tests were conducted to determine whether the pathways were significantly up-regulated in PBMC (Figure 3G).

**Figure 4:**
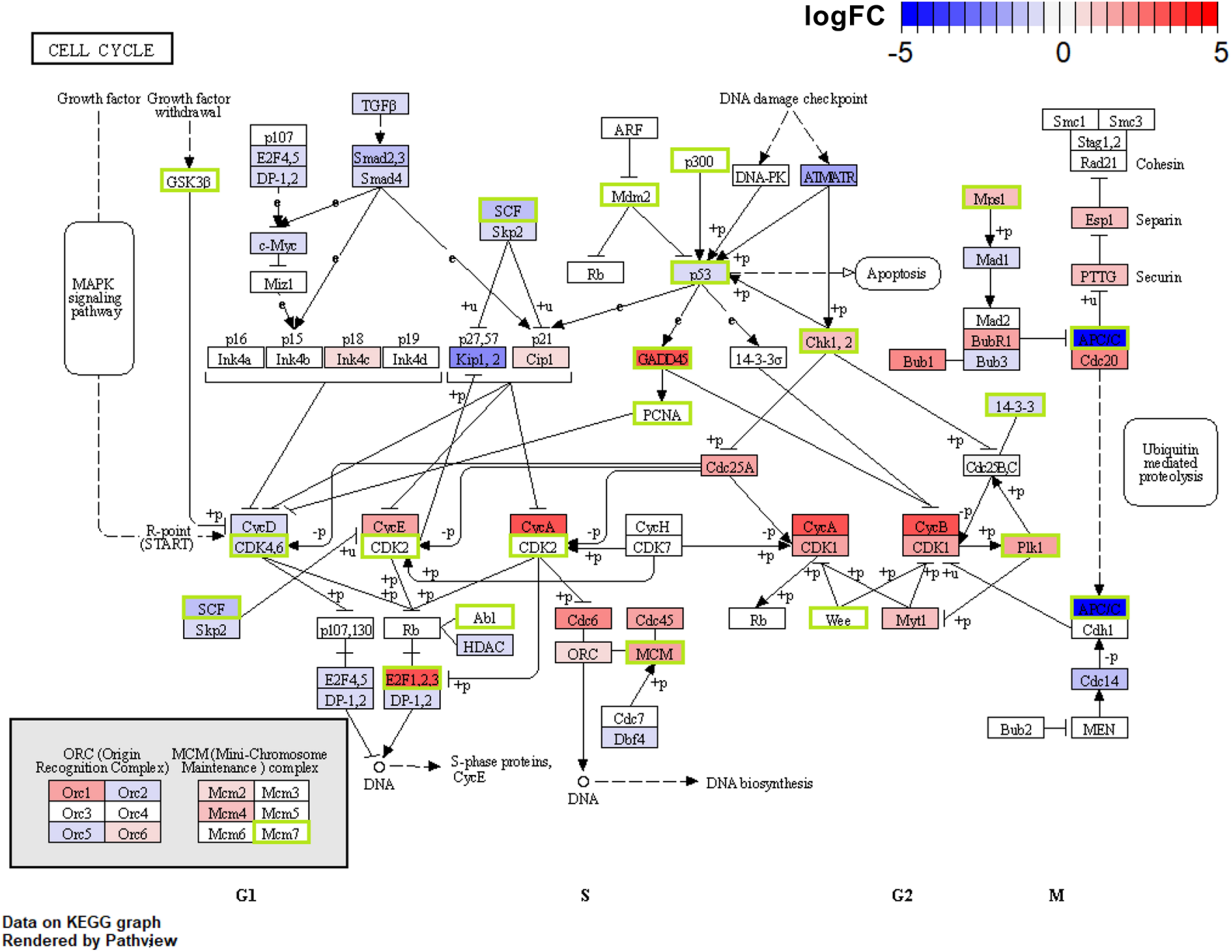
Potential effects of ambroxol in the treatment of COVID-19 in cell cycle pathway in PBMC Proteins shown in the green boxes are the potential targets of ambroxol. Red and blue indicate range of up-regulate or down-regulate of DEGs separately.

As cell cycle and viral carcinogenesis pathways exert high relevance with ambroxol and showed the most significant up-regulation (adj. *P*<0.01), the complete cell cycle diagram was presented in Figure 4, from which we could formulate a hypothesis on how ambroxol affects the cell cycle of PBMC. Ambroxol might bind to CDK2/4/6 and interfere with their functions, thus prevent cells proceed from phase G to phase S1. Additionally, E2F was likewise inhibited by ambroxol to restrain DNA synthesis, S-phase entry, and mitosis. As a consequence, these effects might antagonize SARS-CoV-2-induced inflammatory cell proliferation in PBMC, hence providing protection. Additionally, several targets including as p53, MAPK, etc., might interact with ambroxol (Supplementary Figure S2). These implied further pharmacological effects on PBMC of COVID-19 patients, which was also consistent with the anti-inflammatory effects suggested in the COVID-19 infection pathway.

Enrichment analysis was performed on the down-regulated genes as well. GO and KEGG annotations revealed that the down-regulated genes were prominent in structural constituent of ribosome and immune receptor function, as well as Ribosome and COVID-19 pathways (Supplementary Figure S3A,B). We also examined the signaling pathways on which ambroxol might affect (Supplementary Figure S3C,E). However, because these signaling pathways were down-regulated in COVID-19 patients and we usually assumed that the binding of ambroxol to its target might hinder the target’s activity, we believed that these pathways have limited relevance for the therapy of moderate-to-severe COVID-19.

### Polypharmacology of ambroxol in lung of severe SARS-CoV-2 patients

**Figure 5:**
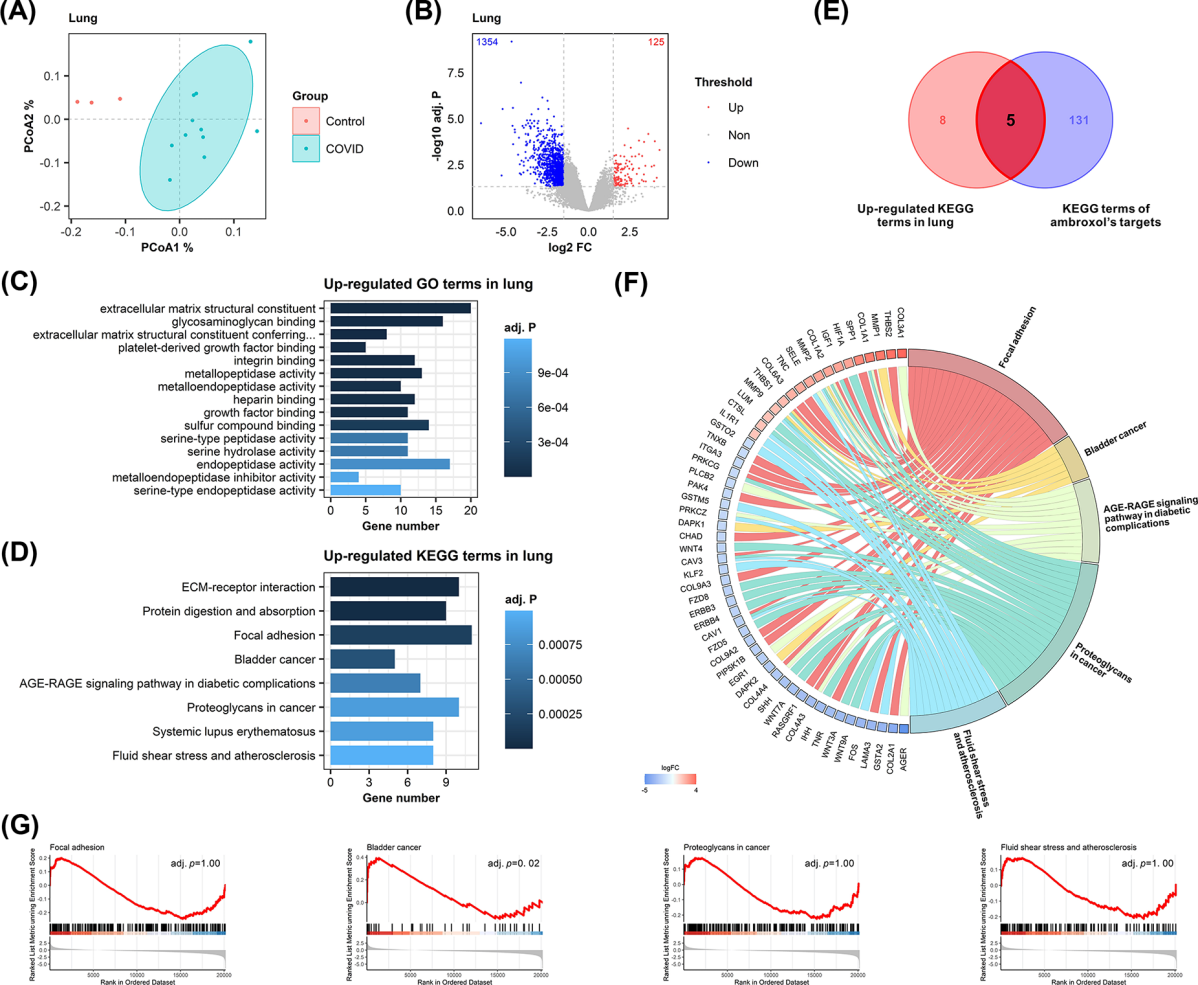
Role of ambroxol in up-regulated pathways in lung tissue (**A**) PCoA diagram of differences in lung tissue between the COVID-19 patients and the healthy controls. (**B**) Volcano plot of DEGs of lung tissue. Genes with adj. *P*<0.05 and |log2FC| > 1 are considered significantly differentially expressed, as shown in upper left and right quadrants. (**C**) GO enrichment results of significantly up-regulated genes in lung tissue. The top 15 significant themes with adj. *P*<0.05 were shown here. (**D**) KEGG enrichment results of significantly up-regulated genes in lung tissue. The top 15 significant themes with adj. *P*<0.05 were shown here. (**E**) Venn diagram of up-regulated KEGG terms in lung tissue and KEGG terms of ambroxol’s targets. (**F**) Chord diagram of up-regulated genes and their corresponding pathways. Color beside gene name indicates range of up-regulate or down-regulate. (**G**) GSEA tests of corresponding up-regulated pathways in lung tissue.

Apart from evaluating the polypharmacology of ambroxol in COVID-19 PBMC, we also investigated the polypharmacology of ambroxol in COVID-19 patients’ lung tissue. Using the same methodologies and criteria, we performed PCoA analysis (Figure 5A) and searched for DEGs using RNA-seq data from postmortem lung tissue samples obtained from COVID-19 patients and those who died of nonpulmonary conditions, identifying 125 significantly up-regulated and 1354 down-regulated genes (Figure 5B). GO and KEGG annotation indicated that the up-regulated genes were predominantly associated with extracellular matrix structural constituent and glycosaminoglycan binding, and ECM-receptor interaction and protein digestion and absorption pathway was most significantly enriched (Figure 5C,D). There were five intersecting routes between up-regulated lung processes and ambroxol’s targets, as indicated by the overlapping pathways (Figure 5E,F). GSEA tests were also conducted to confirm the overall level of variation of the intersecting pathways (Figure 5G).

**Figure 6:**
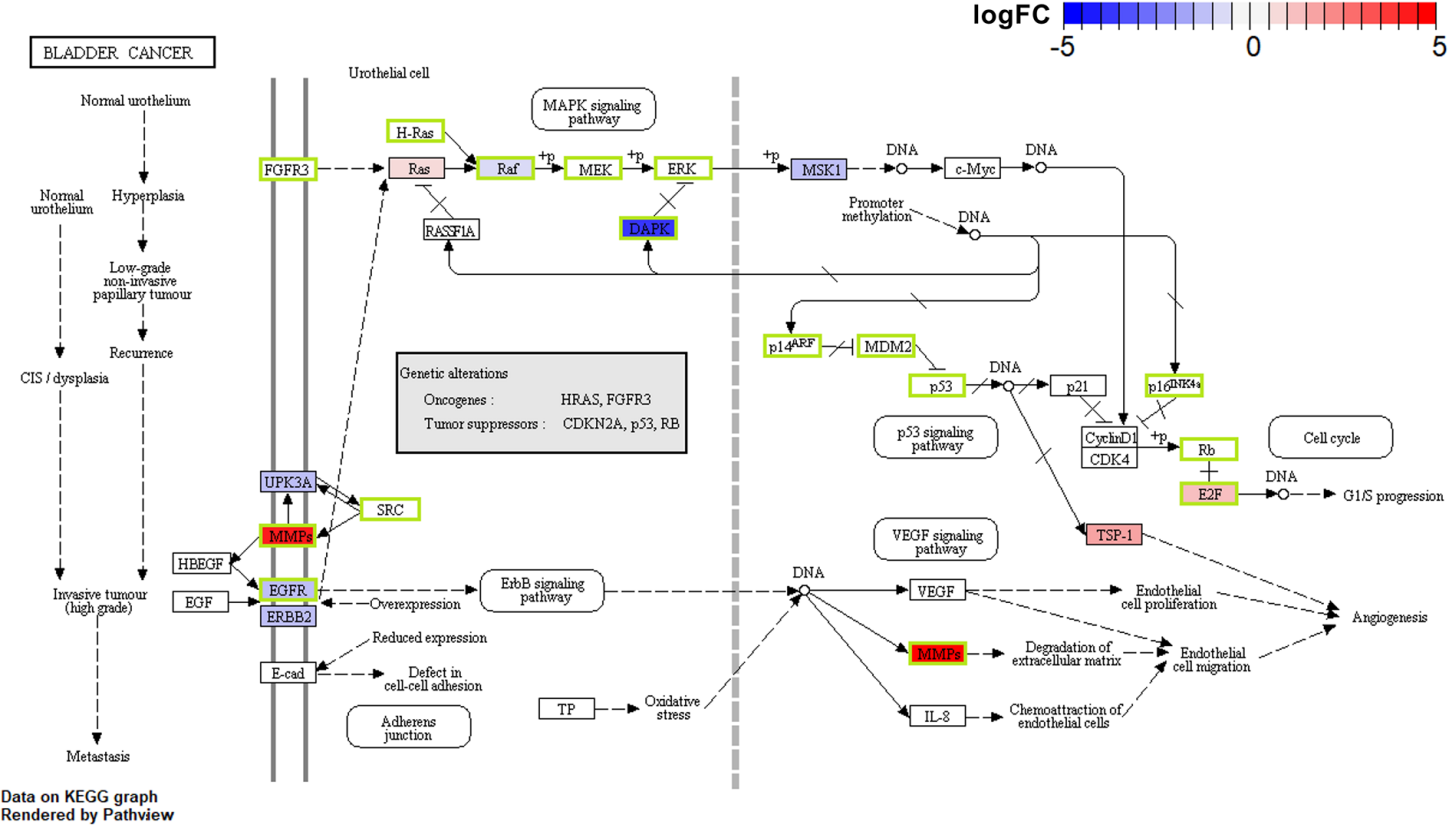
Potential effects of ambroxol in the treatment of COVID-19 in bladder cancer pathway in lung tissue Proteins shown in the green boxes are the potential targets of ambroxol. Red and blue indicate range of up-regulate or down-regulate of DEGs separately.

As referred, Bladder cancer pathway has a rather close relevance with ambroxol in COVID-19 treatment. Expansion of this pathway revealed that matrix metalloproteinases (MMPs) not only functioned as the targets of ambroxol but also as the up-regulated gene. Thus, ambroxol’s interference with MMPs might reduce the degradation of extracellular matrix, diminish endothelial cell proliferation, therefore prevent the increase in airway resistance (Figure 6).

GO analysis of down-regulated DEGs was shown in Supplementary Figure S4A. There was no substantially intersected KEGG pathways in lung tissue according to the previous threshold, indicating that ambroxol had limited effect on aggravating signaling pathways in the lung (Supplementary Figure S4B).

## Discussion

Ambroxol [2-amino-3,5-dibromo-*N*-(*trans*-4-hydroxycyclohexyl) benzylamine], a synthetic derivative of vasicine, is one of the over-the-counter mucolytic drugs that has been used clinically for the treatment of various respiratory diseases [22]. Because of its ability to promote bronchial secretion and clearance [23], as well as inducing surfactant synthesis from alveolar type 2 cells [24], ambroxol has been used as a mucoactive agent and a secretagogue that aids in the reduction of viscid or excessive secretions in diseases, including chronic bronchitis, chronic obstructive pulmonary disease (COPD), cystic fibrosis (CF), bronchiectasis, and asthma [25]. Therefore, intravenous ambroxol can be utilized to treat severe cases in COVID-19. Furthermore, while ambroxol’s pharmacological effects have been extensively studied, its mechanism remains an open question. The presesnt study shed light on its systematic underlying mechanism, particularly in COVID-19 treatment, and complemented its pharmacological effects. Ambroxol was discovered to produce anti-inflammatory and antiviral effects by interacting with targets including NRP-1, EGFR, etc., in multiple signaling pathways.

NRP-1, known to bind furin-cleaved substrates, serves as a host component that significantly potentiates SARS-CoV-2 infectiousness by facilitating viral entry into cells [26]. Besides human angiotensin-converting enzyme 2 (hACE-2) in the previous study [27], we discovered that ambroxol might interact with NRP-1 on the cellular membrane to prevent SARS-CoV-2 entry. In addition, we found the EC_50_ for ambroxol was 135.6 μM in 293T cells and 163.7 μM in A549 cells. NRP-1 is highly expressed in endothelial and epithelial cells of the respiratory and olfactory epithelium [28]. Daly et al. discovered that SARS-CoV-2 used the viral C-end rule (CendR) of Spike (S) protein 1 to bind NRP-1 for attachment and entrance into host cells [29]. Thus, inhibiting this interaction may limit then entrance and infectiousness of SARS-CoV-2.

As severe COVID-19 infection is characterized by a significant inflammatory response, inhibiting the classic inflammatory pathway NF-kB/MAPK has become one of the therapeutic development strategies to alleviate local inflammatory response and ameliorate patient symptoms [30]. NF-κB is a crucial regulatory element involved in the immune-inflammatory response. Activation of NF-κB signaling triggers inflammatory cytokines, which also up-regulates the inducible nitric oxide synthase (iNOS) [31]. Previous studies have found a reduction in both protein expression and phosphorylation of NF-κB and MAPKs signaling pathways in treated with ambroxol, thus decreased the synthesis of IL-1β, IL-6, and TNF-α, as well as the production of superoxide anion, hydrogen peroxide, and nitric oxide, and the release of cellular granular enzymes such as lysozyme [31–33]. Moreover, Yamaya et al. observed that ambroxol decreased RV14 infection in part by lowering ICAM-1 and acidic endosomes via suppressing NF-κB activation [34]. It is quite likely that ambroxol diminishes inflammation by blocking the cascade of cytokines via the NF-κB signaling pathway.

We described the possibility that ambroxol interferes with the function of CDK2/4/6 and E2F. As demonstrated by prior research, CDK4/6 may phosphorylate the retinoblastoma protein, RB1, and the RB-like proteins, RBL1 and RBL2, therefore freeing the E2Fs binding to unphosphorylated RB1 and enabling S-phase entrance [35]. As a result, suppression of these genes may antagonize SARS-CoV-2-induced proliferation in PBMC and exerts ambroxol’s pharmacological impact. The pharmacological function of ambroxol can also be found in the lung tissue of severe COVID-19 patients. Components of the extracellular matrix can be degraded by MMPs in respiratory system of severe COVID-19 patients, resulting in airway remodeling, increased resistance, and permanent pulmonary fibrosis [36]. Therefore, the capacity of ambroxol to limit the effect of MMPs has the potential to improve lung function and prevent excessive hypoxia in patients with severe COVID-19.

According to our results, ambroxol shows a high potency of treating COVID-19 along with its polypharmacological benefits such as mucociliary clearance ability, anti-inflammatory effect, and adequate NRP-1-targeting ability. Importantly, it has been demonstrated that ambroxol interacts with many targets in both PBMC and the lung. Still, it is worthwhile to explore if ambroxol has any effect on SARS-CoV-2’s own proteins, such as spike protein, RNA polymerase, and master protease (Mpro), in order to acquire a better understanding of ambroxol’s effectiveness against SARS-CoV-2. There is also the possibility of combining ambroxol with other antiviral drugs and delivery systems to prolong drug maintenance or lessen adverse effects on normal tissues [37,38]. In conclusion, the present study provided evidence for molecular pathways behind ambroxol’s therapeutic utility in COVID-19 prevention and treatment. Ambroxol is a promising therapeutic candidate against SARS-CoV-2.

## Supplementary Material

Supplementary Figures S1-S4 and Table S1

## Data Availability

Data of all screened human potential protein targets underlying Figure 1 are available in Supplementary Table S1. All other data are available from the corresponding author upon reasonable requests.

## Abbreviations

COVID-19: coronavirus disease 2019
DEG: differentially expression gene
ECM: extracellular matrix
FBS: fetal bovine serum
GEO: Gene Expression Omnibus
GO: Gene Ontology
GSEA: Gene Set Enrichment Analysis
IL: interleukin
KEGG: Kyoto Encyclopedia of Genes and Genomes
MMP: matrix metalloproteinase
NCBI: National Center for Biotechnology Information
NF-κB: nuclear factor kappa B
NRP: neuropilin
PBMC: peripheral blood mononuclear cell
PBST: phosphate buffer solution containing Tween-20
PCoA: principal coordinates analysis
SARS-CoV-2: severe acute respiratory syndrome coronavirus 2
TLR: Toll-like receptor
TPM: transcriptomes per million
