# Supplementary material for: Polypharmacology of ambroxol in the treatment of COVID-19
Source: Biosci Rep. 2023 Feb 27;43(2):BSR20221927. doi: 10.1042/BSR20221927 (PMC9970826; doi:10.1042/BSR20221927)
Supplement: Supplementary Figures S1-S4 and Table S1 [file BSR-2022-1927_supp.pdf]

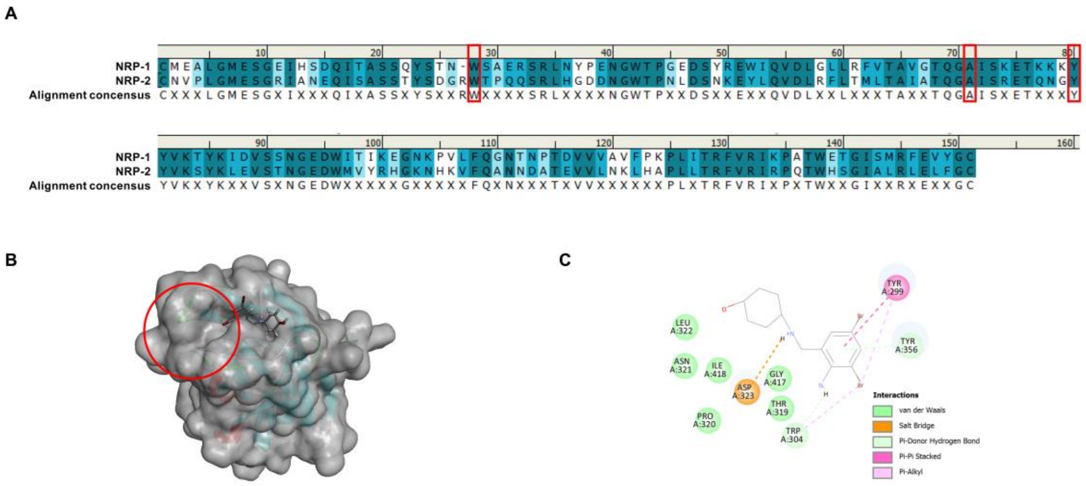

Figure S1. Interaction analysis of anbroxol to NRP-2. (**A**) Similarity alignment of the amino acid sequences of NRP-1 and NRP-2. Red boxes labeled possible key amino acids of NRP-1-ambroxol interaction. (**B**) Possible binding feature of ambroxol with the C1 domain of NRP-2. Ambroxol was shown as sticks with carbon, oxygen and nitrogen colored gray, red and blue, respectively. Secondary structural elements are depicted as ribbons (coils, α-helices; arrows, β-sheets). Color is based on secondary structures (α-helices, red; β-sheets, skyblue; loops, green). (**C**) 2D diagram of molecular interaction of ambroxol with NRP-2.

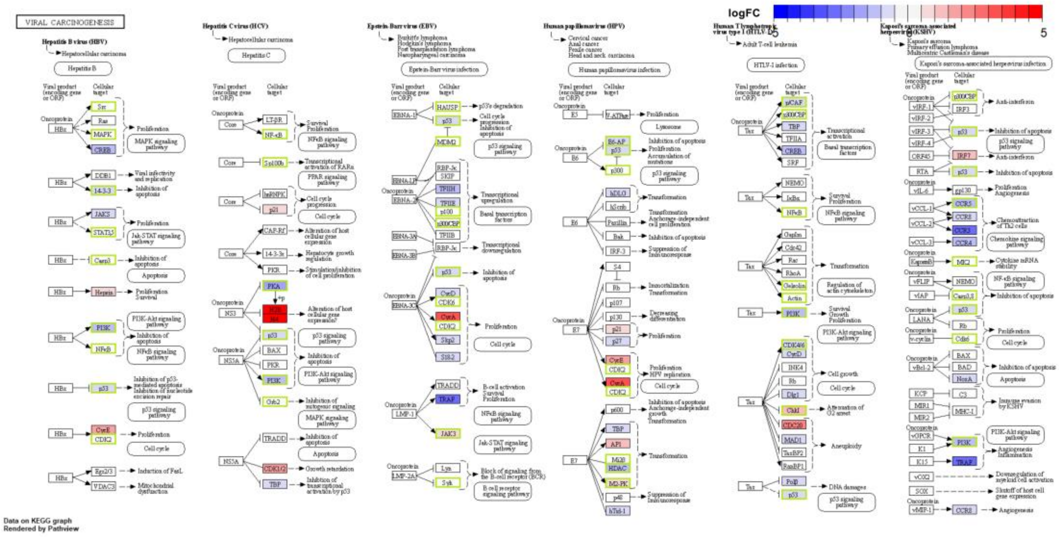

Figure S2. Potential effects of ambroxol in the treatment of covid-19 in Viral carcinogenesis pathway in PBMC. Proteins shown in the green boxes are the potential targets of ambroxol. Red and blue indicates range of up-regulate or down-regulate of DEGs separately.

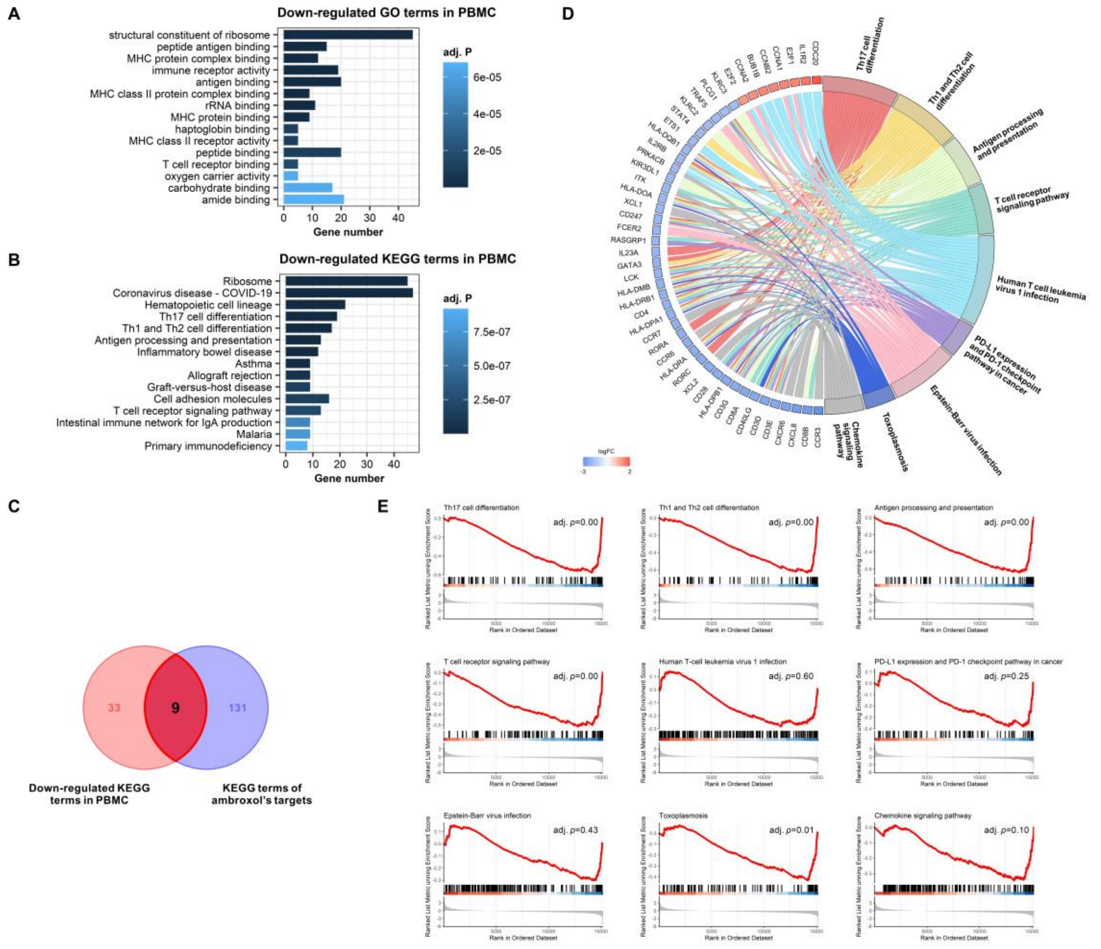

Figure S3. Role of ambroxol in down-regulated pathways in PBMC. (**A**) GO enrichment results of significantly down-regulated genes in PBMC. The top 15 significant themes with adj. P < 0.05 were shown here. (**B**) KEGG enrichment results of significantly down-regulated genes in PBMC. The top 15 significant themes with adj. P < 0.05 were shown here. (**C**) Venn diagram of down-regulated KEGG terms in PBMC and KEGG terms of ambroxol's targets. (**D**) Chord diagram of down-regulated genes and their corresponding pathways. Color beside gene name indicates range of up-regulate or down-regulate expression. (**E**) GSEA tests of corresponding down-regulated pathways in PBMC.

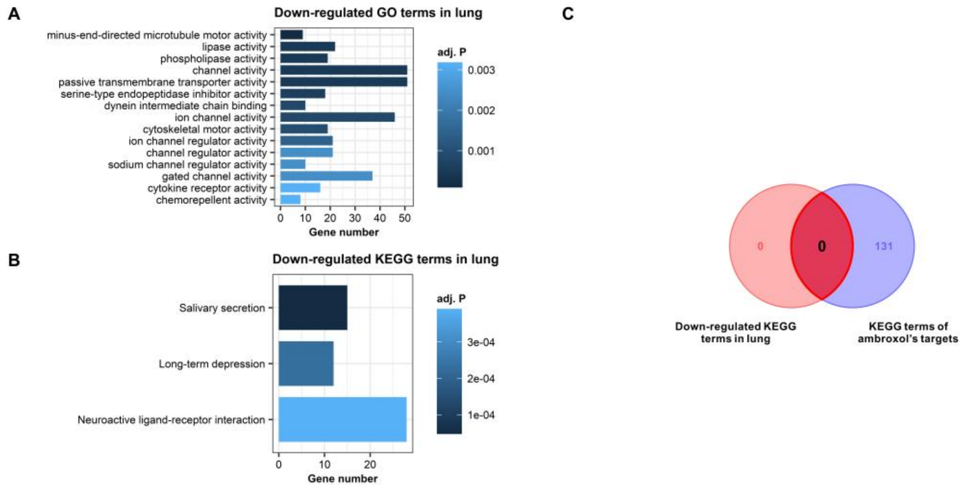

Figure S4. Role of ambroxol in down-regulated pathways in lung tissue. (**A**) GO enrichment results of significantly down-regulated genes in lung tissue. The top 15 significant themes with adj. P < 0.05 were shown here. (**B**) KEGG enrichment results of significantly down-regulated genes in lung tissue. (**C**) Venn diagram of up-regulated KEGG terms in lung tissue and KEGG terms of ambroxol's targets. There is no intersected down-regulated KEGG pathways in lung tissue according to the previous threshold.
